# Rapid Detection of PBP2a in Staphylococci from Shortly Incubated Subcultures of Positive Blood Cultures by an Immunochromatographic Assay

**DOI:** 10.1128/spectrum.00462-21

**Published:** 2021-07-28

**Authors:** Natalia Kolesnik-Goldmann, Elias Bodendoerfer, Kim Röthlin, Sebastian Herren, Frank Imkamp, Martina Marchesi, Stefano Mancini

**Affiliations:** a University of Zurich, Institute of Medical Microbiology, Zurich, Switzerland; Houston Methodist Hospital

**Keywords:** MRSA, PBP2a, blood culture, methicillin resistance, rapid tests

## Abstract

Staphylococcus aureus, as well as coagulase-negative staphylococci (CoNS), can cause a wide range of human infections both in nosocomial and community settings. Βeta-lactams are the antibiotics of choice for the treatment of bloodstream infections (BSI) caused by these microorganisms. Resistance to virtually all β-lactams (also referred to as methicillin resistance) primarily results from the production of an alternative penicillin-binding protein (PBP2a) encoded by the *mecA* gene. While β-lactams are still used as first-line therapy against BSI caused by S. aureus, BSI with CoNS are usually treated with vancomycin due to the high prevalence of methicillin resistance. Rapid detection of methicillin resistance is thus critical for continuation or adjustment of the empirical therapy and therewith to improve the clinical outcome of the patients. The revised version of the immunochromatographic assay PBP2a SA culture colony test (SACCT) is a rapid, inexpensive, and easy method that enables reliable detection of PBP2a in *mecA*-positive staphylococcal isolates after18 to 24 h of incubation. Here, we evaluated the diagnostic performance of the SACCT using primary subcultures of spiked blood cultures after short incubation (4 to 6 h) and established a modified procedure with an equal analytical performance to that of longer-grown cultures. With the proposed method the SACCT can be employed for PBP2a detection from shortly incubated subcultures of clinically relevant staphylococcal isolates, thereby allowing more rapid and effective management of BSI caused by these organisms.

**IMPORTANCE** Antibiotic resistance poses a major threat to health and incurs high economic costs worldwide. Rapid detection of resistance mechanisms can contribute to improving patient care and preventing the dissemination of antimicrobial resistance. Here, we describe a rapid method to detect the most important beta-lactam resistance mechanism (the plasmid-encoded alternative transpeptidase PBP2a) in staphylococcal isolates causing BSI. We show that, using a modified procedure, PBP2a can be reliably detected from primary subcultures of spiked blood cultures after short incubation (4 to 6 h) with a rapid, inexpensive, and simple immunochromatographic test (SACCT). We provide an accurate, inexpensive, and rapid method to facilitate appropriate management and control of infections in patients suffering from invasive staphylococcal infections.

## INTRODUCTION

Staphylococcus aureus is the major human-pathogenic staphylococcal species that can cause a wide range of clinical infections, both in nosocomial and community settings, including infective endocarditis, bacteremia, skin and soft tissue infections, pneumonia, and food poisoning (1). Other Staphylococcus spp., such as Staphylococcus epidermidis and Staphylococcus haemolyticus, are important nosocomial pathogens that can cause device-related infections as well as serious infections in preterm newborns (2, 3). Staphylococcus lugdunensis has been associated with a variety of infections such as endocarditis and soft tissue infections (4). Treatment of staphylococcal infections may be compromised by acquisition of resistance mechanisms toward antibiotics such as β-lactams. Between 2017 and 2019, the rates of methicillin-resistant S. aureus and coagulase-negative staphylococci (CoNS) human isolates in Switzerland were 7.4% and 44.8%, respectively (https://www.anresis.ch). Due to the even lower prevalence of MRSA-invasive human isolates (4.4% in 2017) (5), β-lactams are largely used for empirical antibiotic treatment in case of suspected severe S. aureus infections, while vancomycin is employed for CoNS. Methicillin resistance in staphylococci is primarily due to the acquisition of the *mecA* gene coding for an alternative transpeptidase penicillin-binding protein (PBP2a). With the exception of ceftobiprole and ceftaroline, PBP2a has a very low affinity for almost all β-lactam antibiotics (6). Other mechanisms, such as the *mecA*-homologue *mecC* gene, hyperproduction of β-lactamases in borderline oxacillin-resistant S. aureus (BORSA) and/or point mutations in the PBP3 and/or PBP4 genes in modified S. aureus (MODSA), are, by far, less frequent, especially in Switzerland (7–9). Therefore, detection of PBP2a is, in most cases, sufficient to decide on the continuation or adaptation of the empirical antibiotic treatment of severe staphylococcal infections.

In clinical microbiology laboratories, detection of methicillin resistance is generally based on antimicrobial susceptibility testing (AST) using cefoxitin and/or oxacillin as screening antibiotics (10). These methods require 18 h of incubation in addition to 6 to 24 h for the primary culture. For more rapid detection from clinically relevant biological materials, such as blood cultures, different molecular tests that detect the *mecA* gene directly thereof have been developed (11, 12). However, these tests are expensive, especially taking into account that CoNS are the most common blood culture contaminants that cannot be distinguished from relevant pathogens on the basis of the Gram stain results (13). In addition, false-positive results may be generated from polymicrobial blood cultures containing mixtures of true pathogens, such as S. aureus or S. lugdunensis, which are usually *mecA* negative, and contaminants, which are often *mecA* positive. Phenotypic methods can be alternatively used for detection of the PBP2a protein from primary cultures. The revised version of the immunochromatographic assay developed by Alere (PBP2a SA culture colony test [SACCT]; Abbott Diagnostics Scarborough, Inc., USA) is a rapid, reliable, and easy-to-use test for detection of PBP2a-mediated methicillin resistance in staphylococci (14, 15).

Previous studies have reported that the SACCT displayed superior sensitivity (100%) and the same specificity (100%) in detecting PBP2a-mediated β-lactam resistance in S. aureus, as well as in CoNS isolates, compared to the old version (14, 15). In these studies, the performance of the SACCT has been evaluated with isolates cultivated for 18 to 4 h. This implies that this assay can be employed one workday after the Gram stain identification of staphylococci in native materials at the earliest. However, staphylococci such as S. aureus have high growth rates, and subcultures from biological materials with high bacterial load, such as positive blood cultures, may become visible within 3 to 6 h of incubation (16). In the present study, we evaluated the reliability of the SACCT using primary subcultures of spiked blood cultures after short incubation periods (4 to 6 h) with a collection of 38 S. aureus and 60 CoNS isolates. Furthermore, we investigated the impact of culture preinduction with cefoxitin and reading of the test after 10 min (instead of the recommended 5 min) on the analytical performance of the SACCT. Finally, using an optimized procedure, we conducted a prospective analysis using 112 staphylococcal strains isolated from clinical blood culture flasks.

## RESULTS

In the course of the initial phase of the study, growth of S. aureus primary subcultures of spiked blood culture fluids was, in general, visible after 4 h of incubation, whereas growth of CoNS became apparent after 5 to 6 h (Table S1 in the supplemental material). When subcultures were not preinduced with cefoxitin, 96% (24/25; 95% confidential interval [CI], 88.3% to 100%) of the *mecA*-positive S. aureus strains tested positive upon reading of the SACCT after 5 min (standard) and 10 min. Upon preinduction with cefoxitin, PBP2a was correctly detected in all 25 *mecA*-positive S. aureus isolates already after 5 min. Signal bands were very clear in almost all the cases, irrespective of the reading time or whether bacterial cultures were or were not preinduced with cefoxitin. Without preinduction with cefoxitin, 92% (12/13; 95% CI, 77.3% to 100%) of the *mecA*-negative S. aureus strains correctly tested negative after both 5 and 10 min, while preinduction with cefoxitin induction resulted in a corrected negative result for 13 *mecA*-negative S. aureus strains after 5 and 10 min. Overall, the preinduction with cefoxitin slightly improved the sensitivity, specificity, and robustness of the SACCT for PBP2a detection in S. aureus isolates, while the reading of the results after 10 instead of the recommended 5 min resulted in a few cases in clearer PBP2 bands (Table 1 and Table S1).

**TABLE 1:** Performance of the SACCT with shortly incubated subcultures of spiked blood cultures (after 4 to 6 h)

Species and isolate	No. of isolates	Results for cultures without induction after:						Results for cultures induced with cefoxitin after:					
		5 min incubation			10 min incubation			5 min incubation			10 min incubation		
		Positive	Negative	Indeterminate	Positive	Negative	Indeterminate	Positive	Negative	Indeterminate	Positive	Negative	Indeterminate
*mecA*-positive isolates													
S. aureus	25	24	1	0	24	1	0	25	0	0	25	0	0
S. epidermidis	8	8	0	0	8	0	0	8	0	0	8	0	0
S. haemolyticus	10	9	1	0	10	0	0	9	1	0	10	0	0
S. hominis	8	1	7	0	4	3	1	6	1	1	7	0	1
S. caprae/capitis	4	4	0	0	4	0	0	4	0	0	4	0	0
S. lugdunensis	11	9	2	0	10	0	1	11	0	0	11	0	0
Total no. of isolates (% [95% CI])	66	55 (83.3^*a*^ [74.3–92.3])	11 (16.7)	0	60 (90.9^*a*^ [84–97.8])	4 (6.1)	2 (3)	63 (95.5^*a*^ [90.4–100])	2 (3)	1 (1.5)	65 (98.5^*a*^ [95.5–100])	0	1 (1.5)
*mecA*-negative isolates													
S. aureus *mecC*	3	0	3	0	0	3	0	0	3	0	0	3	3
S. aureus	10	1	9	0	1	9	0	0	10	0	0	10	0
S. epidermidis	5	0	4	1	1	4	0	0	5	0	0	5	0
S. haemolyticus	5	0	4	1	0	3	2	0	5	0	0	5	0
S. hominis	5	3	2	0	2	1	2	0	5	0	0	5	0
S. lugdunensis	4	0	4	0	0	4	0	0	4	0	0	4	0
Total (% [95% CI])	32	4 (12.5)	26 (81.3^*b*^ [67.7–94.8])	2 (6.25)	4 (12.5)	24 (75^*b*^ [60–90])	4 (12.5)	0	32 (100)^*b*^	0	0	32 (100)^*b*^	0

a Percent sensitivity.

b Percent specificity.

When CoNS were not preinduced with cefoxitin, 76% (31/41; 95% CI, 62.4 to 88.7%) of the *mecA*-positive strains tested positive after 5 min. After 10 min, the test was positive for 88% (36/41; 95% CI, 77.8 to 97.8%) of the isolates (Table 1) and resulted, in most cases, in much clearer PBP2a bands (Table S1). For strains preinduced with cefoxitin, 93% (38/41; 95% CI, 84.7% to 100%) tested positive after 5 min and all but one (98%; 40/41; 95% CI, 92.8% to 100%) after 10 min. With the exception of S. hominis, additional 5 min did not affect the intensity of the PBP2a bands. For S. hominis, the preinduction with cefoxitin and the reading after 10 min had a substantial impact on the sensitivity and specificity of the test. While for uninduced methicillin-resistant S. hominis, only 12.5% (1/8; 95% CI, 0 to 35.4%) of the strains were positive after 5 min, 87.5% (7/8; 95% CI, 64.6% to 100%) of the isolates tested positive upon preinduction with cefoxitin and reading after 10 min. Without preinduction with cefoxitin, only 73.7% (14/19; 95% CI, 53.9 to 93.5%) and 63.2% (12/19; 95% CI, 42.4 to 83.9%) of the methicillin-sensitive CoNS isolates tested negative (as expected, the band corresponding to PBP2a did not appear) after 5 and 10 min, respectively. Of note, only 20% (1/5) of the methicillin-sensitive S. hominis isolates tested negative after 5 and 10 min. Upon induction with cefoxitin, all 19 CoNS isolates tested negative both after 5 and 10 min incubation. Overall, both the reading of the test at 10 min and the preinduction with cefoxitin significantly improved the sensitivity (98%), specificity (100%), and robustness of the test for PBP2a detection in CoNS.

Taken together, these results show that the preinduction with cefoxitin and the reading of the test after 10 min allow the detection PBP2a with the SACCT in shortly incubated (4 to 6 h) staphylococcal subcultures with high sensitivity (98.5%; 95% CI, 95.6% to 100%) and specificity (100%). In contrast, following the manufacturer’s instructions (without cefoxitin induction and reading after 5 min), the SACCT does not perform as well as with the modified method presented (sensitivity, 83.3%, 95% CI, 74.3 to 92.3%, and specificity, 81.3%; 95% CI, 67.7 to 94.8%). In view of these findings, a prospective study was conducted with primary subcultures of routine positive blood culture flasks preinduced with cefoxitin and reading of the SACCT after 10 min.

In agreement with the low prevalence of invasive MRSA isolates in Switzerland (4.4% in 2017) (5), in the course of the prospective study, only 2 *mecA*-positive S. aureus strains were isolated, where PBP2a was correctly detected. All 57 *mecA*-negative S. aureus isolates tested negative (Table 2). Bacterial growth after 4 h of incubation was, in general, sufficient, and signal bands from positive samples were very clear (Table S2). Regarding the CoNS, all 37 *mecA*-positive and 16 *mecA*-negative isolates produced concordant and clear test results. Although growth of CoNS was weaker than that of S. aureus strains, visible bacterial lawns appeared for all strains within 6 h of incubation. In conclusion, in applying cefoxitin preinduction and reading at 10 min, the SACCT correctly identified PBP2a in all *mecA*-positive and all *mecA-*negative staphylococcal isolates, thus exhibiting excellent sensitivity (100%), specificity (100%), and robustness (clear positive and negative results).

**TABLE 2:** Performance of the SACCT with shortly incubated subcultures of clinical blood culture bottles (after 4 to 6 h)

Strain and isolate	No. of isolates	No. of positive isolates	No. of negative isolates	No. of indeterminate isolates
*mecA*-positive isolates
S. aureus	2	2	0	0
S. epidermidis	23	23	0	0
S. haemolyticus	9	9	0	0
S. hominis	4	4	0	0
S. caprae/capitis	1	1	0	0
S. lugdunensis	0	0	0	0
Total (%)	39	39 (100)^*a*^	0	0
*mecA*-negative isolates				
S. aureus	57	0	57	0
S. epidermidis	6	0	6	0
S. haemolyticus	0	0	0	0
S. hominis	4	0	4	0
S. caprae/capitis	5	0	5	0
S. lugdunensis	1	0	1	0
Total (%)	73	0	73 (100)^*b*^	0

a Percent sensitivity.

b Percent specificity.

## DISCUSSION

A comprehensive review published in 2017 reported that a variable portion of positive blood cultures (0.6 to 17%) derives from contaminants introduced either during specimen collection or processing, which, to a large extent, consists of CoNS (75%) (17). While S. aureus or S. lugdunensis are always considered true pathogens regardless of the number of the positive blood cultures, CoNS that are isolated from only 1 out 4 blood cultures bottles are generally deemed to be contaminants. Rapid detection of the *mecA* gene or PBP2a from positive blood cultures without prior identification of the staphylococcal isolates would be thus counterproductive, as it would generate unnecessary costs and irrelevant information. The greatest advantage of the method presented here is that the SACCT can be performed after rapid matrix-assisted laser desorption ionization–time of flight mass spectrometry (MALDI-TOF MS) identification of shortly incubated subcultures, and only with those staphylococcal isolates considered clinically relevant. Polymicrobial blood cultures with more than one staphylococcal species are rare and mostly consist of mixtures of a true pathogen, such as S. aureus, and a blood culture contaminant, such as CoNS. In this context, rapid MALDI-TOF MS may fail to detect slower-growing CoNS. Since S. aureus BSI isolates are more commonly methicillin-susceptible than CoNS, the SACCT may therefore generate false-positive results. To minimize this risk, the SACCT could be performed from subcultures of more than one positive blood culture, especially when methicillin-resistant S. aureus is detected. Thus, identical results (most likely reflecting the presence of the same microorganism[s]) from more than one sample would decrease the risk of false-positive results due to undetected polymicrobial cultures resulting from a contamination (in general, considered as such when CoNS are detected in 1 out of 4 blood culture bottles). However, due to the lack of polymicrobial cultures, we could not address this issue in the prospective study.

The retrospective study has shown that the preinduction with cefoxitin and the reading of the test after 10 min (instead of 5 min) significantly improve the sensitivity, specificity, and robustness of the SACCT for CoNS (especially for S. hominis), but not for S. aureus strains. It remains elusive why S. hominis appears to be more problematic than other CoNS. Thus, the use of SAACT without cefoxitin induction should be avoided when S. hominis is identified. In contrast, the preinduction with cefoxitin appears dispensable for PBP2a detection from shortly incubated subcultures of S. aureus-positive blood cultures. We also note that for S. aureus, the time extension from 5 to 10 min before reading the SACCT result may slightly improve the robustness of the test (Table S1 in the supplemental material).

The improved analytical performance in detecting PBP2a of the SACCT compared to the previous version comes at the cost that it can no longer detect PBP2a in *mecC*-positive S. aureus isolates (14, 15). Since the prevalence of *mecC*-positive S. aureus isolates is currently very low, we do not consider this a major drawback. Notwithstanding, this should always be taken in consideration, and conventional AST should always be performed in parallel for clinically relevant isolates in order to identify *mecC* as well as other rare resistance mechanisms (BORSA or MODSA). A major limitation of our study is that the strain collection included only staphylococcal strains isolated from patients in the greater area of Zurich, which might result in a limited genetic diversity. Further studies with larger and more variable strain collections are required to corroborate the robustness of the method presented here.

Overall, we showed that the SACCT can reliably detect PBP2a in shortly incubated (4 to 6 h) staphylococcal subcultures of positive blood cultures, provided that bacterial growth is visible, the subculture is preinduced with cefoxitin, and the test results are read after 10 min. This workflow can be easily implemented in any diagnostic laboratory and allows the generation of clinically relevant information that may influence treatment decision within a few hours from positivity of the blood cultures. At the same time, this analysis can be limited to those strains considered pathogens while disregarding those considered contaminants, thus minimizing unnecessary analyses.

## MATERIALS AND METHODS

### Bacterial strains.

All staphylococcal clinical strains used in this study (Tables 1 and 2) were isolated during 2019 to 2020 in the routine diagnostic laboratory at the Institute of Medical Microbiology, University of Zurich. When more than one clinical isolate originated from the same patient, only the first one was considered.

### MALDI-TOF MS identification.

The isolates were prepared for MALDI-TOF MS by the direct transfer-formic acid method (18). Species identification was performed using the Bruker Biotyper MALDI-TOF MS system (Bruker Corporation, MA, USA) and the database BDAL 9.0.

### Molecular detection of *mecA* and *mecC*.

Detection of the *mecA* and *mecC* genes was done by an in-house PCR as described previously (19) or by whole-genome sequencing (WGS). For WGS, DNA was extracted from fresh subcultures using the DNeasy UltraClean microbial kit (Qiagen, Hilden, Germany). Library preparation was performed using the Qiagen QIAseq FX DNA kit (Qiagen, Hilden, Germany) according to the manufacturer’s recommendations. Sequencing library quality and size distribution were analyzed on a fragment analyzer automated CE system (Advanced Analytical Technologies Inc., Heidelberg, Germany) using the fragment analyzer 474 HS next-generation sequencing (NGS) kit. Sequencing libraries were pooled in equimolar concentrations and sequenced (paired end, 2 × 150 bp) on an Illumina MiSeq platform (Illumina, San Diego CA, USA). Raw sequencing reads (FASTQ) were filtered and trimmed using Trimmomatic (20). For *mecA* and *mecC* detection, FASTQ files were used to query the CARD (21).

### Preparation of simulated spiked blood cultures.

Bacterial isolates were suspended in 0.9% saline solution to obtain a turbidity of 0.5 McFarland (corresponding to approximately 0.2 × 10^8^ CFU/ml) (22). Bacterial suspensions were diluted twice 1:100 in saline solution (resulting in ca. 0.2 × 10^4^ CFU/ml), and 40 μl of the last dilution were used to inoculate 4 ml of aerobic sterile blood culture fluids, resulting in approximately 20 CFU/ml. Blood culture fluids consisted of media and blood from negative blood culture bottles (BacT/Alert FA Plus culture bottles [bioMérieux, Inc., Durham, USA]) after 7 days incubation in the BacT/Alert Virtuo microbial detection system (bioMérieux, Inc., Durham, USA). The spiked blood culture bottles were incubated at 37°C for 18 to 24 h.

### Prospective study.

Positive blood culture bottles (BacT/Alert FA Plus culture bottles labeled as positive by the BacT/Alert Virtuo microbial detection system) containing Gram-positive cocci in clusters as seen on the Gram stain were immediately subcultured on a blood agar plate.

### Immunochromatographic detection of PBP2a.

PBP2a detection from bacterial cultures was performed using the revised version of PBP2a SA culture colony test, namely, the SACCT (Abbott Diagnostics Scarborough, Inc., USA) (23). This test is a rapid immunochromatographic membrane assay that uses highly sensitive recombinant monoclonal antibody fragments (rFabs) to detect the PBP2a protein directly from bacterial isolates. Primary subcultures of spiked blood culture fluids were analyzed with and without prior cefoxitin induction after short incubation periods (4 to 6 h). For the initial evaluation study, a collection of *mecA*-positive and -negative S. aureus isolates (25 and 13, respectively) and CoNS (41 and 19, respectively) was used (Table 1). An in-house PCR (19) or WGS served as the standard method for *mecA* and *mecC* detection. Six drops of spiked blood culture fluid were streaked on two Columbia blood agar plates (Thermo Fisher Scientific Inc.). A cefoxitin disc (i2a; Perols Cedex, France) was placed on the first quadrant of one of the two plates. After 4 to 6 h of incubation at 37°C, bacterial cultures growing on plates with the cefoxitin disc were sampled at the edge of the growth inhibition zone. Results of the SACCT were interpreted by two independent operators after 5 and 10 min. When the signal corresponding to PBP2a was very faint and barely visible, the result was classified as “indeterminate.” For the subsequent prospective study conducted between April and November 2020, all BacT/Alert FA Plus culture bottles with Gram-positive cocci in clusters that became positive during the night and early morning (per patient was considered only the first positive aerobic or anaerobic blood culture) were analyzed (Table 2). Six drops of a positive blood culture were streaked on a blood agar plate, and a cefoxitin disk was placed on the first quadrant. After 4 h of incubation at 37°C, plates were visually inspected, and bacterial isolates present were identified by MALDI-TOF MS followed by analysis with the SACCT. Results were interpreted by two independent operators after 10 min. To mimic the dynamics of our diagnostic laboratory, the SACCT was performed on all S. aureus and S. lugdunensis isolates. CoNS were analyzed only when at least 2 out of 4 blood cultures became positive (Table S2 in the supplemental material). Phenotypic analysis of the isolates conducted by the routine diagnostic laboratory was used as the standard for detection of methicillin resistance (see below).

### Phenotypic detection of methicillin resistance.

Methicillin resistance in staphylococcal isolates was determined by disc diffusion (24) using cefoxitin and moxalactam as screening antibiotics (25).
