# Supplementary material for: Rapid Detection of PBP2a in Staphylococci from Shortly Incubated Subcultures of Positive Blood Cultures by an Immunochromatographic Assay
Source: Microbiol Spectr. 2021 Jul 28;9(1):10.1128/spectrum.00462-21. doi: 10.1128/spectrum.00462-21 (PMC8552594; doi:10.1128/spectrum.00462-21)
Supplement: SUPPLEMENTAL FILE 1 — Supplemental material. Download SPECTRUM00462-21_Supp_1_seq4.docx, DOCX file, 0.05 MB [file spectrum00462-21_supp_1_seq4.docx]

**Table S1. Performance of the SACCT with shortly incubated subcultures of spiked blood culture fluids.**

|  |  |  | **Without induction** | | **Induced with cefoxitin** | |
| --- | --- | --- | --- | --- | --- | --- |
| ***mecA*-positive isolates** | **N. of**  **isolates** | **Growth** | **5 min** | **10 min** | **5 min** | **10 min** |
| ***S. aureus*** | 25 | Good | +++ | +++ | +++ | +++ |
|  |  | Good | +++ | +++ | +++ | +++ |
|  |  | Good | + | +++ | +++ | +++ |
|  |  | Good | +++ | +++ | +++ | +++ |
|  |  | Good | +++ | +++ | +++ | +++ |
|  |  | Good | +++ | +++ | +++ | +++ |
|  |  | Good | +++ | +++ | +++ | +++ |
|  |  | Good | + | ++ | + | +++ |
|  |  | Good | +++ | +++ | +++ | +++ |
|  |  | Good | +++ | +++ | +++ | +++ |
|  |  | Good | +++ | +++ | +++ | +++ |
|  |  | Good | +++ | +++ | +++ | +++ |
|  |  | Good | +++ | +++ | +++ | +++ |
|  |  | Good | +++ | +++ | +++ | +++ |
|  |  | Good | - | - | +++ | +++ |
|  |  | Good | +++ | +++ | +++ | +++ |
|  |  | Good | +++ | +++ | +++ | +++ |
|  |  | Good | +++ | +++ | +++ | +++ |
|  |  | Good | +++ | +++ | +++ | +++ |
|  |  | Good | +++ | +++ | +++ | +++ |
|  |  | Good | +++ | +++ | +++ | +++ |
|  |  | Good | +++ | +++ | +++ | +++ |
|  |  | Good | +++ | +++ | +++ | +++ |
|  |  | Good | +++ | +++ | +++ | +++ |
|  |  | Good | +++ | +++ | +++ | +++ |
| ***S. caprae/capitis*** | 4 | Poor | + | + | +++ | +++ |
|  |  | Good | ++ | ++ | +++ | +++ |
|  |  | Poor | + | + | +++ | +++ |
|  |  | Poor | + | + | +++ | +++ |
| ***S. epidermidis*** | 8 | Good | + | ++ | +++ | +++ |
|  |  | Good | + | +++ | +++ | +++ |
|  |  | Good | ++ | ++ | +++ | +++ |
|  |  | Poor | + | ++ | +++ | +++ |
|  |  | Poor | + | + | +++ | +++ |
|  |  | Poor | + | + | +++ | +++ |
|  |  | Poor | + | ++ | +++ | +++ |
|  |  | Poor | + | +++ | +++ | +++ |
| ***S. haemolyticus*** | 10 | Good | +++ | +++ | +++ | +++ |
|  |  | Poor | + | ++ | +++ | +++ |
|  |  | Poor | ++ | +++ | +++ | +++ |
|  |  | Poor | + | +++ | +++ | +++ |
|  |  | Poor | - | + | - | +++ |
|  |  | Poor | +++ | +++ | +++ | +++ |
|  |  | Poor | +++ | +++ | +++ | +++ |
|  |  | Poor | +++ | +++ | +++ | +++ |
|  |  | Poor | ++ | +++ | ++ | +++ |
|  |  | Poor | + | ++ | ++ | +++ |
| ***S. hominis*** | 8 | Poor | - | - | +++ | +++ |
|  |  | Poor | - | + | + | ++ |
|  |  | Poor | - | +(?) | + | ++ |
|  |  | Poor | - | + | + | ++ |
|  |  | Poor | - | + | + | ++ |
|  |  | Poor | + | + | +++ | +++ |
|  |  | Poor | - | - | +(?) | + |
|  |  | Poor | - | - | - | +(?) |
| ***S. lugdunensis* (n=11)** | 11 | Good | + | ++ | +++ | +++ |
|  |  | Good | + | +++ | +++ | +++ |
|  |  | Good | ++ | ++ | +++ | +++ |
|  |  | Poor | + | ++ | +++ | +++ |
|  |  | Good | ++ | ++ | +++ | +++ |
|  |  | Good | + | + | +++ | +++ |
|  |  | Good | +++ | +++ | +++ | +++ |
|  |  | Good | ++ | ++ | +++ | +++ |
|  |  | Poor | - | +(?) | ++ | +++ |
|  |  | Poor | - | + | ++ | +++ |
|  |  | Poor | + | ++ | +++ | +++ |
| **Total agreement** | **66** |  | **55** | **60** | **63** | **65** |
|  |  |  |  |  |  |  |
| ***mecA*-negative isolates** |  |  |  |  |  |  |
| ***S. aureus mecC*** | 3 | Good | - | - | - | - |
|  |  | Good | - | - | - | - |
|  |  | Good | - | - | - | - |
| ***S. aureus*** | 10 | Good | - | - | - | - |
|  |  | Good | - | - | - | - |
|  |  | Good | - | - | - | - |
|  |  | Good | - | - | - | - |
|  |  | Good | - | - | - | - |
|  |  | Good | - | - | - | - |
|  |  | Good | - | - | - | - |
|  |  | Good | + | + | - | - |
|  |  | Good | - | - | - | - |
|  |  | Good | - | - | - | - |
| ***S. epidermidis*** | 5 | Good | - | - | - | - |
|  |  | Poor | - | - | - | - |
|  |  | Poor | - | - | - | - |
|  |  | Poor | - | - | - | - |
|  |  | Poor | +(?) | + | - | - |
| ***S. haemolyticus*** | 5 | Good | - | - | - | - |
|  |  | Good | - | - | - | - |
|  |  | Good | - | +(?) | - | - |
|  |  | Good | - | - | - | - |
|  |  | Good | +(?) | +(?) | - | - |
| ***S. hominis*** | 5 | Good | - | - | - | - |
|  |  | Poor | +(?) | +(?) | - | - |
|  |  | Poor | +(?) | + | - | - |
|  |  | Poor | +(?) | + | - | - |
|  |  | Poor | - | +(?) | - | - |
| ***S. lugdunensis*** | 4 | Good | - | - | - | - |
|  |  | Good | - | - | - | - |
|  |  | Good | - | - | - | - |
|  |  | Good | - | - | - | - |
| **Total agreement** | **32** |  | **26** | **24** | **32** | **32** |

The green and red backgrounds indicate agreement and disagreement with the expected results, respectively. In the case of positive results, the different green shades reflect the intensity of the band corresponding to PBP2a. The yellow background indicates unclear results. Total agreement indicates the number of concordant results.

**Table S2. Performance of the SACCT with shortly incubated subcultures of clinical blood culture bottles.**

| ***mecA*-positive isolates** | **N. of isolates** | **N. of positive/total blood cultures** | **Growth** | **Results** |
| --- | --- | --- | --- | --- |
| ***S. aureus*** | 2 | 1/4 | Good | ++ |
|  |  | 1/8 | Good | +++ |
| ***S. caprae/capitis*** | 1 | 1/2 | Poor | + |
| ***S. epidermidis*** | 23 | 4/4 | Good | +++ |
|  |  | 1/2 | Good | +++ |
|  |  | 4/4 | Good | +++ |
|  |  | 2/4 | Good | +++ |
|  |  | 2/4 | Good | +++ |
|  |  | 2/2 | Good | +++ |
|  |  | 1/2 | Good | +++ |
|  |  | 4/6 | Good | +++ |
|  |  | 1/1 | Poor | ++ |
|  |  | 1/1 | Good | +++ |
|  |  | 3/4 | Good | +++ |
|  |  | 2/2 | Poor | +++ |
|  |  | 2/2 | Poor | +++ |
|  |  | 2/6 | Good | +++ |
|  |  | 2/4 | Good | +++ |
|  |  | 1/1 | Good | +++ |
|  |  | 1/1 | Good | +++ |
|  |  | 1/2 | Good | +++ |
|  |  | 3/4 | Good | ++ |
|  |  | 2/4 | Good | +++ |
|  |  | 2/4 | Good | +++ |
|  |  | 2/4 | Good | +++ |
|  |  | 3/4 | Good | +++ |
| ***S. haemolyticus*** | 9 | 4/4 | Good | +++ |
|  |  | 2/4 | Good | +++ |
|  |  | 1/4 | Good | +++ |
|  |  | 1/1 | Good | + |
|  |  | 2/4 | Good | +++ |
|  |  | 2/6 | Good | +++ |
|  |  | 2/4 | Good | +++ |
|  |  | 2/2 | Good | +++ |
|  |  | 1/2 | Good | +++ |
| ***S. hominis*** | 4 | 3/4 | Good | +++ |
|  |  | 4/4 | Good | ++ |
|  |  | 2/4 | Good | +++ |
|  |  | 1/2 | Good | +++ |
| **Total agreement** | **39** |  |  |  |
|  |  |  |  |  |
| ***mecA*-negative isolates** | | |  |  |
| ***S. aureus*** | 57 | 4/4 | Good | - |
|  |  | 3/4 | Poor | - |
|  |  | 4/4 | Good | - |
|  |  | 2/4 | Good | - |
|  |  | 2/4 | Good | - |
|  |  | 4/4 | Good | - |
|  |  | 2/4 | Good | - |
|  |  | 3/4 | Good | - |
|  |  | 1/4 | Good | - |
|  |  | 3/4 | Good | - |
|  |  | 4/4 | Good | - |
|  |  | 4/4 | Good | - |
|  |  | 3/4 | Good | - |
|  |  | 1/4 | Good | - |
|  |  | 2/4 | Good | - |
|  |  | 1/4 | Good | - |
|  |  | 1/4 | Good | - |
|  |  | 1/4 | Good | - |
|  |  | 3/4 | Good | - |
|  |  | 2/2 | Good | - |
|  |  | 4/4 | Good | - |
|  |  | 2/2 | Good | - |
|  |  | 3/4 | Poor | - |
|  |  | 2/4 | Good | - |
|  |  | 2/8 | Good | - |
|  |  | 4/6 | Good | - |
|  |  | 1/4 | Good | - |
|  |  | 4/4 | Good | - |
|  |  | 4/4 | Good | - |
|  |  | 3/4 | Good | - |
|  |  | 3/4 | Good | - |
|  |  | 8/8 | Good | - |
|  |  | 6/8 | Good | - |
|  |  | 4/4 | Good | - |
|  |  | 3/4 | Good | - |
|  |  | 2/2 | Good | - |
|  |  | 1/4 | Good | - |
|  |  | 1/4 | Good | - |
|  |  | 1/4 | Good | - |
|  |  | 1/4 | Good | - |
|  |  | 4/4 | Good | - |
|  |  | 2/4 | Good | - |
|  |  | 2/4 | Good | - |
|  |  | 4/4 | Good | - |
|  |  | 3/4 | Good | - |
|  |  | 8/8 | Good | - |
|  |  | 1/4 | Good | - |
|  |  | 4/4 | Good | - |
|  |  | 4/4 | Good | - |
|  |  | 4/4 | Good | - |
|  |  | 4/4 | Good | - |
|  |  | 4/4 | Good | - |
|  |  | 2/4 | Good | - |
|  |  | 4/4 | Good | - |
|  |  | 1/4 | Good | - |
|  |  | 6/8 | Good | - |
|  |  | 4/4 | Good | - |
| ***S. caprae/capitis*** | 5 | 2/4 | Good | - |
|  |  | 3/4 | Good | - |
|  |  | 2/4 | Good | - |
|  |  | 2/2 | Good | - |
|  |  | 1/2 | Good | - |
| ***S. epidermidis*** | 6 | 3/4 | Good | - |
|  |  | 2/3 | Good | - |
|  |  | 2/4 | Good | - |
|  |  | 2/4 | Good | - |
|  |  | 4/4 | Good | - |
|  |  | 1/2 | Good | - |
| ***S. hominis*** | 4 | 2/4 | Poor | - |
|  |  | 2/4 | Good | - |
|  |  | 2/4 | Good | - |
|  |  | 1/2 | Good | - |
| ***S. lugdunensis*** | 1 | 1/2 | Good | - |
| **Total agreement** | **73** |  |  |  |

The green background indicates agreement with the expected results. In the case of positive results, the different green shades reflect the intensity of the band corresponding to PBP2a. Total agreement indicates the number of concordant results.
